# BetaHCG secretion by a pulmonary adenocarcinoma

**DOI:** 10.1186/1477-7819-11-228

**Published:** 2013-09-14

**Authors:** Cécile Vicier, Emeline Tabouret, Agnès Tallet, Anthony Gonçalves, Bruno Chetaille, Patrice Viens, Anne Madroszyk

**Affiliations:** 1 Department of Medical Oncology, Institut Paoli-Calmettes, 13009 Marseille, France; 2 Aix-Marseille Université, 13001 Marseille, France; 3 Radiotherapy Department, Institut Paoli-Calmettes, 13009 Marseille, France; 4 Institut Paoli-Calmettes, Anatomopathology, 13009 Marseille, France

**Keywords:** BetaHCG, Non-small-cell lung cancer, Paraneoplastic syndromes, Ectopic pregnancy

## Abstract

We report a rare case of metastatic non-small-cell lung cancer in a 43-year-old woman with a history of smoking. The tumor secreted human chorionic gonadotropin and its beta subunit (BetaHCG). The patient presented with amenorrhea, a positive pregnancy test and chest pain. A physical examination and investigations revealed no pregnancy, and it was determined that a paraneoplastic syndrome stemming from a pulmonary tumor was responsible for the secretion of BetaHCG. This secretion decreased with tumor response to chemotherapy. Only a few reports of paraneoplastic BetaHCG secretion can be found in the literature for several different cancers.

## Background

The lung cancer mortality rate has been declining in men, especially for those between age 35 and 44 years of age. The decline mirrors a significant reduction in men’s smoking habits. However, lung cancer mortality in women has been increasing, especially in younger women. Moreover, recent studies have shown that female smokers have a 10-year reduced lifespan compared with their non-smoking counterparts [1]. Cancer diagnosis can be made from primary tumor, metastatic lesions or, less commonly, paraneoplastic syndromes, primarily encountered in lung cancer. Paraneoplastic syndromes (neoplasm-associated alterations resulting from damage to organs or tissues that are remote from the tumor site) are rare. They occur in 8% of cancer patients and frequently develop with advanced disease but may appear earlier than symptoms of the primary tumor itself, leading to a cancer diagnosis [2]. We report here the case of a young woman with a history of smoking who presented with a beta human chorionic gonadotropin (BetaHCG) paraneoplastic secretion, an indicator of non-small-cell lung cancer. We subsequently present a comprehensive literature review and discuss prognosis and therapeutic options.

## Case presentation

A 43-year-old woman (gravida 2, para 1, abortion 1 in 2010) presented with amenorrhea, nausea, chest pain and asthenia. Her medical history included a diagnosis of hepatitis C 2 years ago, weaned heroin and cocaine addiction and active tobacco addiction. She had smoked two packs of cigarettes daily for a period of 20 years and drank two glasses of wine per day. She had no treatment or allergy.

When she presented at the hospital, pregnancy was suspected because of a 2-month history of amenorrhea and lack of consistent use of contraception. A qualitative BetaHCG test was positive. Pelvic examination revealed a normal sized uterus and normal adnexum. She had neither bleeding nor pelvic pain.

**Figure 1:**
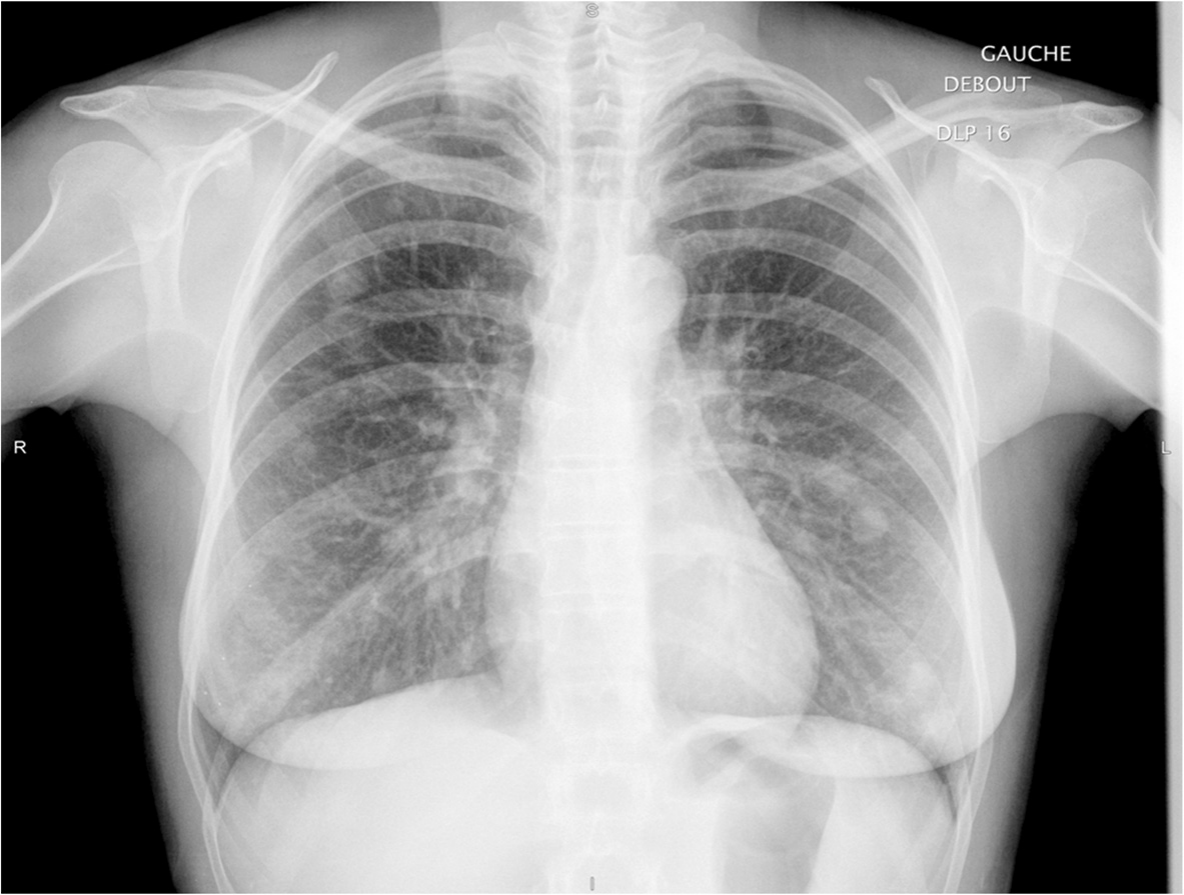
Chest radiography at the diagnosis.

Serums concentrations of BetaHCG, taken 2 days apart, were 1,135 and 1,180 UI/l (negative value < 5 UI/l). Because of the elevated serum BetaHCG level, with an abnormal increase, the patient underwent a pelvic ultrasound, which revealed an empty uterine cavity and normal ovaries. Uterine curettage was performed and revealed no chorionic villi. Therefore, a laparoscopy was performed, which revealed a normal abdominal cavity, eliminating an ectopic pregnancy diagnosis. A germ cell tumor was then suspected. Despite two cycles of methotrexate, the patient’s BetaHCG concentrations increased (3,393 UI/l). At the same time, the patient’s chest pain worsened, leading to the performance of a chest radiography (Figure 1), which revealed many lesions in both lungs.

**Figure 2:**
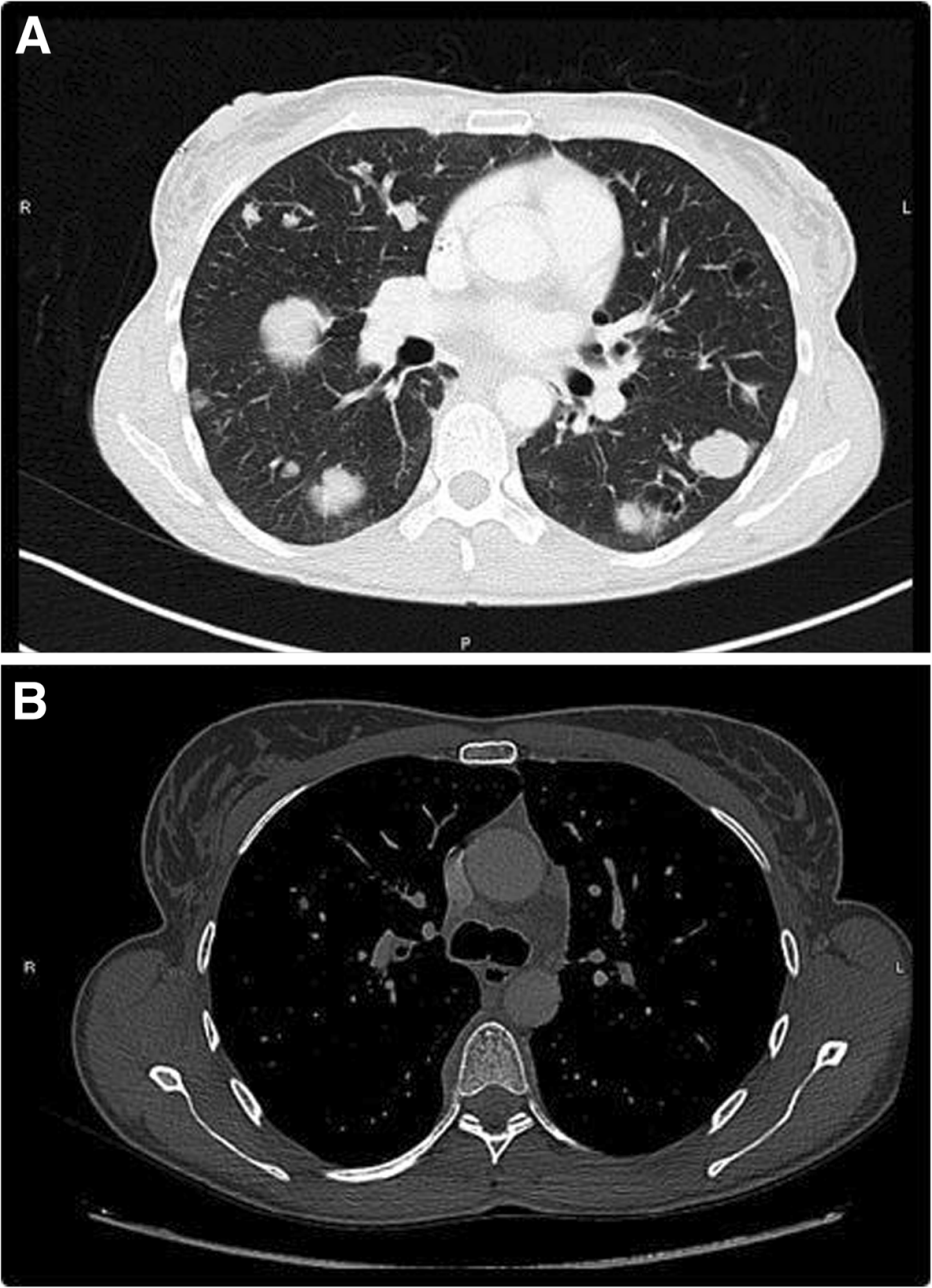
**Computed tomography scan at the initial diagnosis. (A)** Parenchymal sequence. **(B)** Mediastinal sequence.

A computed tomography scan (Figure 2) confirmed multiple pulmonary lesions. The most significant lesion was on the right lung (23 mm) with ipsilateral hilar node. A bone scan showed a paravertebral mass, which explained the chest pain. The bronchoscopy was macroscopically normal. A lung biopsy was performed. Histological examination revealed a poorly differentiated carcinoma, AE1-AE3 and CK7 positive, CK20, TTF1 and OCT3-4 negative, with cytoplasmic vacuoles positive to anti-HCG antibody. No *EGFR* or *KRAS* mutation or ALK rearrangement was found, but an amplification of the ALK gene was observed in 22% of the analyzed cells. Moreover, blood tests were performed to evaluate the presence of tumor markers and showed normal levels of carbohydrate antigen 19–9 (CA19.9), neuron specific enolase and alpha-fetoprotein. We noted a slightly increased level of carcinoembrionic antigen (ACE = 8 ng/ml, negative value < 5 ng/ml) and cytokeratin 19 fragment (Cyfra21 = 4.32 ng/ml, negative value < 3 ng/ml).

The paravertebral mass was rapidly irradiated, and a cisplatin-based chemotherapy was initiated. After three courses of a cisplatin-vinorelbine regimen, a computed tomography scan showed progressive disease in the lung, and BetaHCG concentrations continued to increase markedly. Chemotherapy was therefore modified; treatment with docetaxel was initiated. After four courses, a partial tumor response was obtained in the lung, but brain metastases appeared. The BetaHCG concentration dropped to 436 UI/L. The patient received whole-brain radiotherapy and two additional cycles of docetaxel. Once the disease was stabilized, she received an oral 1-year maintenance treatment of erlotinib but ultimately experienced tumor progression and died from complications of the disease.

## Discussion

This young female patient with a BetaHCG-secreting non-small-cell lung cancer was initially believed to be pregnant. We noticed an elevated BetaHCG concentration and amenorrhea. However, due to increasing chest pain, pathologic thoracic radiography, a computed tomography scan and lung biopsies were performed. We eliminated a choriocarcinoma based on histological examinations, which showed a poorly differentiated carcinoma (AE1-AE3 and CK7 positive, CK20, TTF1 and OCT3-4 negative). Moreover, after her abortion in 2010, she had the return of her menstrual cycles for 1 year, the pelvic examination was normal and uterine curettage revealed no chorionic villi. The BetaHCG secretion was suspected to be due to a paraneoplastic syndrome associated with the primary tumor. Indeed, the BetaHCG concentration as well as all lung lesions decreased in response to second-line chemotherapy, indicating that the BetaHCG secretion was due to a paraneoplastic syndrome related to her primary lung tumor.

Outside of pregnancy, a high level of BetaHCG may appear in molar pregnancies, choriocarcinomas and germ cell tumors such as testicular tumors or ovarian tumors [3]. In these cases, the BetaHCG secretion is considered a paraneoplastic syndrome.

Paraneoplastic syndromes are an extremely diverse group of clinical aberrations that are associated with non-invasive actions of tumors. They develop mainly in hematologic malignancies and, to a lesser extent, with thymoma and small-cell lung cancer. Although they may affect a variety of systems, their target is the endocrine system [2]. Their precise pathophysiologic mechanism is still unknown but has been suggested to be ectopic secretion by tumor cells. Moreover, paraneoplastic syndromes are known to be present in about 10% of lung cancer cases [4]. Cases in the literature have reported several Cushing syndromes associated with lung cancers and other malignancies [5-7].

Based on reports in the medical literature, paraneoplastic syndromes involving BetaHCG secretion are very uncommon. Paraneoplastic BetaHCG secretion has been reported in cases of squamous cell carcinoma of the head and neck [8], phyllode tumors of the breast [9], clear cell renal cell carcinoma [10], and leiomysarcoma [11].

BetaHCG secretion is rarely associated with lung cancer. Paraneoplastic syndrome has been described in a squamous cell lung cancer [12], large-cell lung carcinoma (BetaHCG secretion = 206 mIU/l) [13] and three cases of pulmonary adenocarcinoma [14,15]. In the two last cases of pulmonary adenocarcinoma, the BetaHCG level was 13 UI/l and 19 UI/l, respectively. These levels are significantly lower than the level observed in our patient. Moreover, these studies examined the interpretation of BetaHCG testing for women of childbearing age before treatment and inclusion in clinical trials. Our report focused on a different clinical problem. Given a high level of HCG, the possibility of pregnancy must first be eliminated, delaying tumor diagnosis and initiation of appropriate therapy.

Moreover, this case emphasizes the diversity of lung cancer presentations and the diagnostic complexity of tumors revealed by a paraneoplastic syndrome. Secondary amenorrhea and a high level of BetaHCG secretion are not always associated with a germinal tumor and may be related to a paraneoplastic syndrome, suggesting immediate and extensive investigation in the context of no pregnancy.

## Conclusion

We diagnosed a lung adenocarcinoma in a young woman based on the presence of BetaHCG secretion; this is considered a rare paraneoplastic syndrome. The patient had exceptionally high levels of BetaHCG compared with other cases reported in the literature.

## Consent

Written informed consent was obtained from the patient’s family for publication of this report and any accompanying images.

## Abbreviations

BetaHCG: Beta human chorionic gonadotropin.

## Competing interests

The authors declare that they have no competing interests.

## Authors’ contributions

CV acquired and analyzed data and drafted the manuscript. ET, AT, AG, BC and PV participated in the acquisition of data and helped to draft the manuscript. AM supervised this case report, participated in the acquisition of data and helped to draft the manuscript. All authors read and approved the final manuscript.
